# Supplementary material for: Research on the relationship between the scattering contribution and physical factors of the reference radiation regulated by ISO 4037–1
Source: PLoS One. 2022 Dec 29;17(12):e0279188. doi: 10.1371/journal.pone.0279188 (PMC9799293; doi:10.1371/journal.pone.0279188)
Supplement: Attachment — Submitted filename: Notes.docx [file pone.0279188.s003.docx]

Notes:

1- Introduction:  Is well organized; however, it should be a summary of the literature review. Additionally, there is no comprehensive description of why the method is used?
2- Equations (2) and (3) need references.
3- In Table 1 how did choose the value of the input vector?
4- In the construction process of the LS-SVM relational model, the stage of adjusting the LS-SVM model is not clear and needs to explain (Fig 2).
5- The paper should be rewritten as the following:1-introduction 2-methodology 3-results-4-discussion 5-Conclusion.
6- You need to correct many grammatical mistakes.
