# Supplementary material for: Research on the relationship between the scattering contribution and physical factors of the reference radiation regulated by ISO 4037–1
Source: PLoS One. 2022 Dec 29;17(12):e0279188. doi: 10.1371/journal.pone.0279188 (PMC9799293; doi:10.1371/journal.pone.0279188)
Supplement: Attachment — Submitted filename: Response to Reviewers.docx [file pone.0279188.s004.docx]

Dear Reviewers,

Thank you very much for your time involved in reviewing the manuscript and your very encouraging comments on the merits.

We also appreciate your clear and detailed feedback and hope that the explanation has fully addressed all of your concerns. In the remainder of this letter, we discuss each of your comments individually along with our corresponding responses.

To facilitate this discussion, we first retype your comments in italic font and then present our responses to the comments.

We would like to take this opportunity to thank you for all your time involved and this great opportunity for us to improve the manuscript. We hope you will find this revised version satisfactory.

Yours sincerely,

Peng Feng

**Responses List to Reviewer 1**

- **Comment 1:**

*Introduction: Is well organized; however, it should be a summary of the literature review. Additionally, there is no comprehensive description of why the method is used?*

- **Response 1:**

Thanks for your comment. In the revision, we revised the content of the introduction part. In the last two paragraphs, we added the reason why LSSVM method was used for modeling and PSO algorithm was used for optimization.

- **Comment 2:**

*Equations (2) and (3) need references.*

- **Response 2:**

In the revision, reference 'Mathematical statistics with applications' for equations (2) and (3) was added.

- **Comment 3:**

*In Table 1 how did choose the value of the input vector?*

- **Response 3:**

Thanks for your comment. Table 1 shows the value of the input vector under different radioactive sources. If the univariate control method is used to select the values of *D_3_*, *D_2_*, *H*, and *W* in Table 1, 9^4^ groups of simulation experiments are required. The workload of simulation is huge and difficult to achieve. In order to effectively select the eigenvectors in the modeling and reduce the number of simulation experiments, the orthogonal experiment method is adopted here: According to the orthogonal table L_81_ (9^4^) of 4 factors and 9 levels of each factor, 81 sets of input vector groups are obtained.

In the revision, we supplement the method of selecting the input vector，in the paragraph below Table 1.

- **Comment 4:**

*In the construction process of the LS-SVM relational model, the stage of adjusting the LS-SVM model is not clear and needs to explain (Fig 2).*

- **Response 4:**

Thanks for the comment. In the revision, we have revised and supplemented the modeling process of LSSVM, in the paragraph below Figure 2.

In the stage of adjusting LS-SVM model, the parameters *γ* and *σ^2^* need to be adjusted. Because the model needs to be established is low in complexity and few parameters, the grid search method is used here: setting the value range of parameters *γ* and *σ^2^*, trying every possibility through loop traversal, and finally getting the best combination of parameters.

- **Comment 5:**

*The paper should be rewritten as the following:1-introduction 2-methodology 3-results-4-discussion 5-Conclusion.*

- **Response 5:**

Thank you for the comment. In the revision, we adjusted the structure of the paper into four parts: 1-Introduction; 2-Methods; 3-Results and discussion; 4-Conclusion.

- **Comment 6:**

*You need to correct many grammatical mistakes.*

- **Response 6:**

Thank you for the detailed review. We have carefully and thoroughly proofread the manuscript to correct all the grammar and typos.

**Responses List to Reviewer 2**

- **Comment 1:**

*The topic and Objective set out in the work is clear. The methodology used is also appropriate and the final results have been clearly spelt out. The work clearly contribute some missing knowledge as far as the use of ISO 4037-1:2019 is concerned*

- **Response 1:**

Thank you for your positive comments.
